# Inadequate glucose control in type 2 diabetes is associated with impaired lung function and systemic inflammation: a cross-sectional study

**DOI:** 10.1186/1471-2466-10-38

**Published:** 2010-07-26

**Authors:** Rodolfo J Dennis, Dario Maldonado, Maria X Rojas, Pablo Aschner, Martin Rondón, Laura Charry, Alejandro Casas

**Affiliations:** 1 Department of Research, Fundación Cardioinfantil-Instituto de Cardiología, Calle Kra 13b No 163-85, Bogotá, Colombia; 2 Fundación Neumológica Colombiana, Calle 163ª Kra 13b, Bogotá, Colombia; 3 Departament of Clinical Epidemiology and Bioestatistics, Kra 7 No 40-62, Pontificia Universidad Javeriana, Bogotá, Colombia; 4 Asociación Colombiana de Diabetes, Calle 39ª Bis No 14-28, Bogotá, Colombia

## Abstract

**Background:**

Inadequate glucose control may be simultaneously associated with inflammation and decreased lung function in type 2 diabetes. We evaluated if lung function is worse in patients with inadequate glucose control, and if inflammatory markers are simultaneously increased in these subjects.

**Methods:**

Subjects were selected at the Colombian Diabetes Association Center in Bogotá. Pulmonary function tests were performed and mean residual values were obtained for forced expiratory volume (FEV_1)_, forced vital capacity (FVC) and FEV_1_/FVC, with predicted values based on those derived by Hankinson et al. for Mexican-Americans. Multiple least-squares regression was used to adjust for differences in known determinants of lung function. We measured blood levels of glycosylated hemoglobin (HBA_1c_), interleukin 6 (IL-6), tumor necrosis factor (TNF-α), fibrinogen, ferritin, and C-reactive protein (C-RP).

**Results:**

495 diabetic patients were studied, out of which 352 had inadequate control (HBA_1c _> 7%). After adjusting for known determinants of lung function, those with inadequate control had lower FEV_1 _(-75.4 mL, IC95%: -92, -59; P < 0.0001) and FVC (-121 mL, IC95%: -134, -108; P < 0,0001) mean residuals, and higher FEV_1_/FVC (0.013%, IC95%: 0.009, 0.018, P < 0.0001) residuals than those with adequate control, as well as increased levels of all inflammatory markers (P < 0.05), with the exception of IL-6.

**Conclusions:**

Subjects with type 2 diabetes and inadequate control had lower FVC and FEV_1 _than predicted and than those of subjects with adequate control. It is postulated that poorer pulmonary function may be associated with increased levels of inflammatory mediators.

## Background

The association of type 2 diabetes with decreased lung function (measured by known determinants of pulmonary disability and death, such as FEV_1_), has received renewed attention [1,2]. Inflammation, microangiopathy, and alterations in lung matrix proteins may all play a role. The first mechanism tends to unify the impairment seen in patients with emphysema with that seen in diabetes and cardiovascular disease; in this context, both type 2 diabetes and chronic lung disease, such as COPD, have been associated with increased levels of low-grade acute and chronic systemic inflammatory mediators and inflammatory markers [3-5]. Until recently, however, few studies had explored lung function among subgroups of diabetic subjects. It is likely that persistent inadequate glucose control over time may alter regulation of inflammatory pathways that are involved in the impairment of lung function [2,6].

It is still unknown if inadequate glucose control in type 2 diabetes is simultaneously associated with a decrease in lung function and increased systemic levels of inflammatory markers. In this study, we compared type 2 diabetes subjects based on their glucose control profile (adequate vs. inadequate) and assessed differences in lung function (spirometry) and blood levels of five known systemic inflammatory markers.

## Methods

This was a cross-sectional study. The sampling frame from which patients were selected was the Colombian Diabetes Association (ACD), a private institution in Bogota, Colombia, that provides basic and specialized ambulatory care for diabetic patients of all socio-economic strata. Patients need not be referred with any special degree of severity or case-mix. We invited to participate in the study both incident and prevalent diabetes patients of either sex, who were seen at the ACD between July 2005 and September 2007. Inclusion criteria were age between 35 and 65 years, and medical diagnosis of type 2 diabetes confirmed at ACM. The Latin-American Diabetes Association (ALAD) definition for type 2 diabetes (fasting glycemia equal to or greater than 126 mgr/dL, glycemia equal to or greater than 200 mgr/dL after a glucose load during a glucose tolerance test, or casual glucose blood levels of 200 mgr/dL or more), is the standardized definition used in the ACD [7]. We excluded patients that met any of the following criteria: pregnancy, previous diagnosis of rheumatoid arthritis or collagen disease; cystic fibrosis, lung cancer or tuberculosis; thoracic, lung, or heart surgery; or deformities that would preclude conducting a reliable spirometric procedure.

The study protocol was approved by the Clinical Research Ethics Committee at Fundación Cardioinfantil in Bogotá.

### Sample size

Based on previous results comparing diabetic patients against controls, we expected mean residual values larger than -60 mL for the FEV_1 _[2] and mean standard deviations between 100 ml and 250 ml in adults over 21 years old [8]. With a type I error probability of 5% and a power of 80%, we estimated a required sample size required of 500 subjects. The study included 495 subjects.

### Data collection

A respiratory questionnaire, previously translated into Colombian Spanish, and validated in other already published research projects [9], was used for screening. Once a case of type 2 diabetes was deemed as potentially eligible, the subject was invited to participate and to give informed consent. Baseline information was collected by interview following a standardized data collection form. The participant was then given an appointment for a second visit the next day for blood sampling to measure fasting glucose, HBA_1c_, levels of circulating inflammatory mediators and markers (IL-6, TNF-α, Fibrinogen, Ferritin, C-RP). Spirometry was also performed in the second visit, as well as height, weight (barefoot and with lightweight indoor clothing), and body-mass index measurements. The spirometer used (Vitalograph, UK) met all American Thoracic Society recommended criteria [10].

Blood samples were taken after a minimum of 6 hours fasting (except insulin-dependent diabetics) using vacuum tubes, centrifuged and processed on a daily basis for glucose fasting levels, HBA1c, fibrinogen and ferritin, following standard daily laboratory operating procedures. A set of serum samples were stored at -20°C for a maximum of two months for other analyses. TNF-α, IL-6, and high sensitivity C-RP were measured by a solid phase, enzyme labeled, chemiluminescent sequential inmunometric assay method, using the Immulite 1000 analyzer (EURO/DPC Ltd, Llanberis, UK). A total of seven runs in batches were carried out during the course of the study. Each run for C-RP, TNF and IL-6 was processed in duplicate, and met standard specific control assays provided by the manufacturer, two for TNF-α and IL-6 (high and low), and three for C-RP (high, intermediate and low).

### Data management and statistical analysis

Data collected was recorded in a previously designed database using ACCESS (Office 2000). All statistical analyses were performed with SAS software programs (SAS Institute, NC). To assess the association of diabetes control as measured with HBA_1c _levels and mean systemic values of inflammatory markers, we categorized HBA_1c _values into equal or less than 7% and greater than 7%, and tested for significance with the *t-test*. We selected this cutoff point *a priori *because it is usually selected in clinical practice to discriminate between appropriate and inappropriate control [11], although some patients may be optimally controlled at slightly higher HbA1c values. A *p *value < 0.05 on a two-sided test was considered significant. Bi-variate analyses included the χ^2 ^test for categorical/nominal data, and the independent t-test or its non-parametric equivalent. Predicted FEV_1_, FVC and FEV_1_/FVC values adjusted for age, sex and height were obtained for every subject, based on the prediction equations obtained by Hankinson et al for Mexican-Americans [12], which have provided good fit to Colombian subjects, based on previous studies by our group [13].

Residual values (observed minus predicted) were obtained for FEV_1_, FVC and FEV_1_/FVC in each subject, negative values representing lower than expected pulmonary function. We also used least-squares multiple linear regression models to further adjust mean residual values for smoking history (current, past, none), body mass index, exposure to indoor wood-smoke inhalation, to assess the significance of interaction terms, and the inclusion of inflammation markers. We also looked at the association between glucose control, lung function and inflammatory markers, by stratifying HBA_1c _values into quintiles, and tested for statistical significance with ANOVA.

## Results

### Subject characteristics

We screened 1888 subjects with history of type 2 diabetes and 495 were finally included in the study. The most frequent cause for exclusion was age (outside the stipulated inclusion criteria); past history of TB, lung fibrosis, lung cancer, and thoracic trauma or surgery. Of the 495 studied type 2 diabetic subjects, 352 (71%) were classified as having inadequate control (HBA_1c _> 7%). Table 1 shows the baseline characteristics of both groups. There were no clinical or statistically significant differences in sex, age, height, weight, exposure to cigarette smoking or indoor wood smoke inhalation, chronic lung disease or past clinical history of important cardiovascular co-morbidity. Those with inadequate glucose control, however, had longer disease duration (9.07 years) than those with adequate glucose control (6.6 years, *P *= 0.0002).

**Table 1:** SUBJECT CHARACTERISTICS (n (%), mean ± sd)

VARIABLE	INADEQUATE CONTROL (n = 352)	ADEQUATE CONTROL (n = 143)	*p V*ALUE
MALE SEX	169 (48.01)	75 (52.45)	0.3714
AGE (years)	53.21 ± 7.81	54.53 ± 7.61	0.0829
35-45	64 (18.18)	19 (13.29)	0.1037
46-55	140 (39.77)	54 (37.76)	
56-65	148 (42.05)	70 (48.95)	
HEIGHT (centimeters)	160.60 ± 10.40	161.42 ± 9.16	0.3865
WEIGHT (kilograms)	72.82 ± 14.43	73.80 ± 13.13	0.4689
SMOKING HISTORY			0.8750
non smoker	151 (42.90)	64 (44.76)	
past smoker	152 (43.18)	58 (50.56)	
current smoker	49 (13.92)	21 (14.69)	
cigarettes/day	5.70 ± 5.86	7.14 ± 6.83	0.4026
median (p_25_-p_75_)	3.0 (2-7)	4.0 (2-10)	
EXPOSURE TO DUST, LIFETIME	113 (32.10)	43 (30.07)	0.6594
EXPOSURE TO GASES, LIFETIME	48 (13.64)	22 (15.38)	0.6133
EXPOSURE TO WOODSMOKE, LIFETIME	100 (28.41)	32 (22.38)	0.1694
CHRONIC BRONCHITIS	8 (2.27)	3 (2.10)	0.9049
LUNG EMPHYSEMA	2 (0.57)	0 (0.00)	0.3659
ASTHMA	8 (2.27)	4 (2.80)	0.7312
PULMONARY EMBOLISM	0 (0.00)	0 (0.00)	

Subjects with inadequate glucose control had lower, unadjusted, FEV_1 _(- 90 mL) and FVC (-150 ml) that did not reach statistical significance, and a somewhat higher FEV_1_/FVC ratio (table 2). In general, there were no differences in FEV1 and FVC between subjects with adequate and inadequate glucose control (table 2). Subjects with inadequate glucose control had lower FVC percent predicted values (below 70%) (*p *= 0.02). There were no differences between groups in the number of subjects that would have been categorized as stage 1 obstruction (GOLD) based on FEV_1_/FVC ratio < 70% (table 2).

**Table 2:** PULMONARY FUNCTION (n (%), mean ± sd)

VARIABLE	**INADEQUATE CONTROL HBA**_**1c **_**>7% (n = 352)**	ADEQUATE CONTROL (n = 143)	*p *VALUE
FEV_1 _(liters)	2.68 ± 0.73	2.77 ± 0.69	0.2080
FVC (liters)	3.36 ± 0.94	3.51 ± 0.86	0.0853
FEV_1_/FVC (%)	80.57 ± 6.50	79.21 ± 6.90	0.0447
FEV_1 _< 70% EXPECTED VALUE (*)	15 (4.26)	2 (1.40)	0.1133
FVC < 70% EXPECTED VALUE (*)	13 (3.60)	0 (0.00)	0.0200
FEV_1_/FVC < 70% EXPECTED VALUE (*)	17 (4.83)	11 (7.69)	0.2119

(*) Expected based on: Hankinson JL, Odencrantz JR, Fedan KB. Spirometric reference values from a sample of the general US population. Am J Respir Crit Care Med 1999; 159: 179-187.

With respect to mean systemic values of inflammatory markers (table 3), TNF-α, fibrinogen, ferritin, and C-RP were significantly higher in those with inadequate glucose control than in those with adequate glucose control (*p *< 0.05)

**Table 3:** INFLAMMATORY MARKERS (mean ± sd)

MARKER	**INADEQUATE CONTROL HBA**_**1c **_**>7% (n = 352)**	ADEQUATE CONTROL (n = 143)	*p *VALUE
C-RP (mg/L)	2.75 ± 5.82	1.52 ± 2.01	0.0005
FERRITIN (ug/L)	163.05 ± 150.24	130.90 ± 114.87	0.0109
FIBRINOGEN (mg/dL)	444.09 ± 116.99	422.68 ± 98.23	0.0389
IL6 (pg/mL)	3.91 ± 3.65	3.56 ± 2.65	0.2399
TNF (pg/mL)	9.03 ± 5.35	7.98 ± 5.01	0.0390

### Relationship between glucose control and pulmonary function

Table 4 shows the differences in pulmonary function between subjects with adequate and inadequate control after statistical adjustment for differences in anthropometric characteristics and risk factor exposure (smoking and wood smoke). Mean residual values for FVC were significantly lower in those with inadequate glucose control than in those with adequate control (FVC difference -121.2 mL; 95%CI: -134.1, -108.4; *p *< 0.0001). Similar findings were seen in FEV_1_: those with inadequate glucose control had lower values than those with adequate control (difference -75.4 mL; 95%CI: -92.2, -58.6, *p *< 0.0001). Those subjects with inadequate control also had greater values of FEV_1_/FVC, (difference: 0.013; 95%CI: 0.009, 0.018, *p *< 0.0001).

**Table 4:** MEAN RESIDUAL PULMONARY FUNCTION (*) AND STRATIFICATION BY SMOKING HISTORY (**)

	INADEQUATE CONTROL		ADEQUATE CONTROL		DIFFERENCE IN MEAN RESIDUALS			
	n	Mean	n	Mean		95% CI		
						LL	HL	*p *Value
**rFEV**_**1**_	**352**	**-164.9**	**143**	**-89.5**	**-75.4**	**-92.2**	**-58.6**	**< 0.0001**
Smoking History								
Non-smoker	151	-137.6	64	-57.1	-80.5	-95.9	-65.1	< 0.0001
Past	152	-181.5	58	-119.3	-62.2	-101.2	-23.0	0.0029
Current	49	-197.3	21	-105.8	-91.5	-173.6	-9.4	0.0194
**rFVC**	**352**	**-247.3**	**143**	**-126.1**	**-121.2**	**-134.1**	**-108.4**	**< 0.0001**
Smoking History								
Non-smoker	151	-192.4	64	-130.8	-61.6	-74.0	-49.2	< 0.0001
Past	152	-294.1	58	-136.1	-158.0	-191.5	-124.4	< 0.0001
Current	49	-271.3	21	-83.7	-187.7	-245.4	-129.9	< 0.0001
**rFEV**_**1**_**/FVC**	**352**	**1.021**	**143**	**1.008**	**0.013**	**0.009**	**0.018**	**< 0.0001**
Smoking history								
Non-smoker	151	1.024	64	1.023	0.001	-0.006	0.008	0.7473
Past	152	1.023	58	0.997	0.026	0.020	0.033	< 0.0001
Current	49	1.007	21	0.991	0.015	0.001	0.032	0.0184

(*) Values in bold are mean residuals for FEV_1_, FVC and FEV_1_/FVC adjusted for differences in age, height, sex, and smoking history.

(**) Values stratified by smoking history are mean residuals adjusted for differences in age, height, and sex.

CI: Confidence interval. LL: lower limit; HL: higher limit.

Table 4 also shows mean residual values stratified by categories of smoking history. There was a tendency to have lower mean residuals with past or present exposure to cigarette smoke, especially in those with inadequate glucose control. No interaction term between BMI, cigarette smoking status, and glucose control in their joint effect on lung function was significant (data not shown). The inclusion of all five inflammation markers to the models did not significantly change results. The only marker significantly associated with residual lung function, after adjustment for all other variables in the models, was C-RP (p = 0.04). Similarly, time elapsed since diabetes diagnosis did not affect point estimates for mean residual values of FEV_1_, FVC or FEV_1_/FVC.

Table 5 shows residual lung function and inflammation marker values by quintiles of HBA_1c _levels. There was a trend towards lower lung function, especially for FVC, and increased levels of inflammation as HBA_1c _levels increased, especially for C-RP, ferritin and fibrinogen.

**Table 5:** QUINTILES OF HBA_1c _VALUES AND RELATION WITH RESIDUAL LUNG FUNCTION (*) AND MEAN INFLAMMATORY MARKER LEVELS

	1	2	3	4	5	
	< = 6.60	6.61-7.50	7.51 - 8.60	8.61 - 10.40	> 10.40	
	n = 107	n = 92	n = 103	n = 97	n = 96	*p *VALUE
**rFEV**_**1 **_**(ml)**	-82.73	-96.05	-130.78	-224.63	-186.30	0.0848
**rFVC (ml)**	-128.72	-133.01	-219.96	-314.23	-270.11	0.0324
**C-RP**	1.55	1.94	1.94	3.71	2.94	0.0155
**FERRITIN**	119.58	146.24	150.96	193.65	161.43	0.0056
**FIBRINOGEN**	424.89	420.60	444.27	438.56	461.67	0.0830
**IL-6**	3.55	3.71	3.88	4.03	3.88	0.8806
**TNF**	8.08	8.31	8.67	9.48	9.16	0.3050

(*) Mean residuals for FEV_1 _and FVC adjusted by differences in age, height, sex, and smoking history.

C-RP: C-reactive protein; TNF: Tumor necrosis factor.

## Discussion

Results of this study show that those diabetic subjects with inadequate glucose control over the past several weeks as measured with HBA_1c_, have lower pulmonary function than those with adequate control. These differences were not explained by usual determinants of lung function such as age, height, sex, cigarette smoking or other lifetime exposures. Subjects with inadequate control also had significantly higher levels of inflammatory markers (TNF-α, ferritin, fibrinogen, and C-RP), although, there is no clear dose-effect relation. We selected these markers, including IL-6, because increased levels of these inflammatory cytokines (IL-6 and TNF-α), as well as downstream proteins such as C-RP, ferritin and fibrinogen, have been implicated in the inflammatory response seen in COPD and diabetes.

The limitations of our approach should be discussed. Although selection bias is a possibility, we minimized it by selecting all consecutive cases with diabetes with no particular severity of disease, duration, or case-mix. Confounding bias is not likely in our study, as we adjusted the results for known determinants of lung function, such as age, height and sex, as well as smoking history. Persistent confounding by smoking may be possible given that we did not quantify the intensity of lifetime exposure (i.e. pack-years). However, the likelihood of this is low, because the intensity of current cigarette exposure seemed to be lower in those with inadequate control. Although those diabetic subjects with poorer control also had longer disease duration, adjustment for time elapsed from diagnosis did not change results. Given that we assessed diabetes duration by patient history, however, we cannot exclude persistent confounding and non-differential misclassification as a possible explanation for this finding. Finally, by taking both incident and prevalent cases of diabetes, our results may be confounded by the effects of treatment and by survivor bias, but both biases, if present, would have tended to decrease the magnitude of the effect sizes seen in this study. Classification bias is not likely to have altered our results, as all subjects met pre-determined criteria for diabetes, and both the definition of control (HBA_1c_) and cut-off point selected to define inadequate control (greater than 7%) were established *a priori. *Some recall bias may have been possible with respect to past medical history and respiratory exposures, but, even if present, these are more likely to have been non-differential between those with adequate and inadequate glucose control (subjects were not aware of the hypothesis under study).

From a clinical perspective, the importance of changes in lung function has usually been judged as the rate of change over time, derived from observational cohorts or clinical trials. Thus, it is difficult to fairly assess the significance of the magnitude of change observed in this study, given the cross-sectional approach. This study confirms previous findings of decreased lung function in diabetic subjects and in those with insulin resistance [2,6,14]. Davis et al and McKeever et al recently reported decreased lung function in diabetic subjects, and convincingly showed that this decrease is associated with inadequate diabetes control, which is consistent with our study results [6,15]. However, our study goes further to show that some inflammatory markers are elevated in patients with simultaneous inadequate control and decreased lung function, suggesting a potential association. Mechanisms related to chronic hyperglycemia may involve both the formation of oxygen radicals and their effect on pulmonary vasculature or the alveolar-capillary membrane, and the secondary effect of systemic inflammation seen in diabetic patients [16-19]; both mechanisms need not be mutually exclusive. The second mechanism tends to equate the impairment seen in patients with emphysema with that seen in diabetes and cardiovascular disease, through inflammatory mediators [20]. In addition to the wealth of literature available on endothelial damage and cardiovascular events, recent studies have shown that systemic inflammation markers such as ferritin [3], fibrinogen [21,22], and C-RP are increased in subjects with type 2 diabetes as indicators of prognosis, as well as inflammation mediators associated with insulin resistance such as IL-1, IL-6, and TNF-α [23].

It should be highlighted that the impairment seen in our study is more consistent with a restrictive lung disorder. Although not a uniform finding, this has been previously described in other studies [1,24,25]. This suggests a causal pathway (or pathways) for inflammation different from that in emphysema and COPD. Alteration of matrix proteins seen in DM due to the formation of advanced glycosylation end products and subsequent inflammation in the lungs [26] has been suggested, and may provide such a pathway.

## Conclusions

Diabetic subjects with inadequate glucose control have lower pulmonary function than those with adequate control, a difference not explained by usual determinants of lung function. Similarly, those subjects with inadequate control also had significantly higher levels of inflammation markers (TNF-α, Ferritin, Fibrinogen, and C-RP), suggesting a potential association. Our results need to be confirmed by longitudinal studies (given that diabetic control is a time-dependent variable). Hopefully, it can also be shown that better glucose control can result not only in no further decrease in lung function but also in attenuation of the inflammatory response. Our findings, however, should make clinicians more aware of this association and add to the necessity of advising diabetic patients of the need for adequate glucose control.

## Competing interests

The authors declare that they have no competing interests.

## Authors' contributions

RD conceived the study and had primary responsibility for design, overall planning, and manuscript drafting. DM participated in the design, data analysis and helped with interpretation. MXR participated in the design, planning, coordinated data gathering, and helped draft the manuscript. PA participated in the design, data gathering and helped with interpretation. MR participated in the design of the study and performed the statistical analysis. LC participated in the design, data gathering, and helped draft the manuscript. AC participated in the design, planning, and helped with interpretation. All authors read and approved the final manuscript.

## Pre-publication history

The pre-publication history for this paper can be accessed here:

http://www.biomedcentral.com/1471-2466/10/38/prepub
